# The Anti-Inflammatory Effect of Human Telomerase-Derived Peptide on *P. gingivalis* Lipopolysaccharide-Induced Inflammatory Cytokine Production and Its Mechanism in Human Dental Pulp Cells

**DOI:** 10.1155/2015/385127

**Published:** 2015-10-28

**Authors:** Yoo-Jin Ko, Kil-Young Kwon, Kee-Yeon Kum, Woo-Cheol Lee, Seung-Ho Baek, Mo K. Kang, Won-Jun Shon

**Affiliations:** ^1^Department of Conservative Dentistry, Dental Research Institute and School of Dentistry, Seoul National University, Jongno-gu, Yeongun-dong 275-1, Seoul 110-749, Republic of Korea; ^2^Department of Family Medicine, Eulji University School of Medicine, Joong-gu, Yongdu-dong 143-5, Daejeon, Republic of Korea; ^3^School of Dentistry, David Geffen School of Medicine, Jonsson Comprehensive Cancer Center, UCLA, Los Angeles, CA 90095, USA

## Abstract

*Porphyromonas gingivalis* is considered with inducing pulpal inflammation and has lipopolysaccharide (LPS) as an inflammatory stimulator. GV1001 peptide has anticancer and anti-inflammation activity due to inhibiting activation of signaling molecules after penetration into the various types of cells. Therefore, this study examined inhibitory effect of GV1001 on dental pulp cells (hDPCs) stimulated by *P. gingivalis* LPS. The intracellular distribution of GV1001 was analyzed by confocal microscopy. Real-time RT-PCR was performed to determine the expression levels of TNF-*α* and IL-6 cytokines. The role of signaling by MAP kinases (ERK and p38) was explored using Western blot analysis. The effect of GV1001 peptide on hDPCs viability was measured by MTT assay. GV1001 was predominantly located in hDPC cytoplasm. The peptide inhibited *P. gingivalis* LPS-induced TNF-*α* and IL-6 production in hDPCs without significant cytotoxicity. Furthermore, GV1001 treatment markedly inhibited the phosphorylation of MAP kinases (ERK and p38) in LPS-stimulated hDPCs. GV1001 may prevent *P. gingivalis* LPS-induced inflammation of apical tissue. Also, these findings provide mechanistic insight into how GV1001 peptide causes anti-inflammatory actions in LPS-stimulated pulpitis without significantly affecting cell viability.

## 1. Introduction

Dentin pulp complex injuries are often induced by invasion of microorganisms and their components via dentinal tubules towards the pulp. Caries, cracks, fractures, and leakage from restorations provide pathways for microorganisms and their toxins to enter the pulp. Odontogenic infections are generally caused by polymicrobial and dominated by anaerobic bacteria [1]. The response of the pulpal irritation is inflammation and eventually pulp necrosis may occur. The inflammation can spread to the surrounding alveolar bone and cause periapical pathosis. In this process, bacterial lipopolysaccharides (LPSs) play a potential role in several responses to pulpal infection. Lipopolysaccharide (LPS) can induce the expression of proinflammatory cytokines and chemokines such as TNF-*α* and IL-6 and elicit the innate immune response in dental pulp cells (DPCs) [2].

Signaling pathways initiated by engagement of toll-like receptors (TLRs), such as TLR2 and TLR4, by bacterial products lead to enhanced transcription of genes responsible for the expression of cytokines, chemokines, adhesion molecules, and other mediators of the inflammatory response associated with bacterial infection. Of note, the activation of mitogen-activated protein kinases (MAPKs) is important in the production of inflammatory cytokines by LPS stimulation [3]. The MAPK family includes extracellular-signal-related protein kinase (ERK), c-JUN N-terminal kinase/stress-activated protein kinases (JNK/SAP) and p38MAPK [4]. The MAPK signaling pathway is involved in various kinds of cellular processes including differentiation, development, proliferation, and survival, as well as cell death, depending on cell type and stimulus [5, 6]. Pulpal p38MAPK signaling is activated by LPS stimulation during the induction of local proinflammatory response [7–9].

Telomeres are specialized structures at the ends of chromosomes that have a role in protecting the chromosome ends from DNA repair and degradation [10]. Telomerase is a cellular reverse transcriptase (TERT, telomerase reverse transcriptase) which prevents premature telomere attrition and maintains normal length and function [11]. Human reverse transcriptase subunit of telomerase (hTERT) has become an attractive target for cancer vaccines due to it being expressed in 85–90% of human cancer tissues, whereas it is almost never expressed in normal tissues [12]. GV1001 peptide, which is a peptide corresponding to amino acids 611–626 of hTERT (EARPALLTSRLRFIPK), has been developed as a vaccine against various cancers and has been reported to have the ability to penetrate into various cells, including cancer cell lines and primary blood cells [13]. GV1001 was found to localize predominantly in the cytoplasm and could successfully deliver macromolecules such as proteins, DNA, and siRNA into cells [13]. Because of this novel pharmaceutical potential and cell-penetrating ability, as well as its own anticancer activity, GV1001 peptide is very promising for use in the medical field. Here, we observed that this peptide could also penetrate into human dental pulp stem cells and, furthermore, that it had a self-anti-inflammatory effect without affecting cell viability.

The purpose of this study was to evaluate the cell-penetrating function of GV1001 peptide in human dental pulp cells (hDPC) and to investigate the anti-inflammatory effect of GV1001 and its related mechanism in* P. gingivalis* LPS-induced inflammation through regression of inflammatory cytokine production.

## 2. Materials and Methods

### 2.1. Synthesis of Peptides

All of the peptides used in this study were synthesized by the Fmoc- (9-fluorenylmethoxycarbonyl-) based solid-phase method and characterized by Peptron Inc. (Daejeon, Korea). The purities of all peptides used in this study were greater than 95%, as determined by high-performance liquid chromatography.

### 2.2. Cells and Cultivation

This study was approved by the Seoul National University Dental Hospital Institutional Review Board. The impacted third molars of human adults were collected from 18- to 22-year-old patients after obtaining informed consent. The isolated dental pulp was cut into small pieces and digested in a solution of 3 mg mL^−1^ type I collagenase and 4 mg mL^−1^ dispase for 30–60 min at 37°C (Sigma Aldrich, St. Louis, MO, USA). Subsequently, the solution was filtered through a 70 mm cell strainer (Becton/Dickinson, Franklin Lakes, NJ, USA). The single-cell suspensions were seeded in 35 or 60 mm culture dishes and maintained in a culture media consisting of *α*-minimum essential medium (*α*-MEM; Invitrogen, Carlsbad, CA, USA) supplemented with 15% fetal bovine serum (FBS; Gibco-BRL, Life Technologies Inc., Gaithersburg, MD, USA), 0.292 mg mL^−1^ glutamine (Invitrogen), 100 units mL^−1^ penicillin G, 100 mg mL^−1^ streptomycin, and 50 mg mL^−1^ ascorbic acid (Sigma). The cells were incubated in a humidified atmosphere containing 5% CO_2_ at 37°C and cells between 3 and 4 passages were used in the following experiments.

### 2.3. Bacterial Species and LPS Extraction

*Porphyromonas gingivalis* ATCC 33277 was purchased from the American Type Culture Collection (ATCC; Manassas, VA) and cultured with brain heart infusion broth (BHI; BD Bioscience, Sparks, MD, USA) supplemented with hemin (1 *μ*g mL^−1^) and vitamin K (0.2 *μ*g mL^−1^) in anaerobic conditions at 37°C. Lipopolysaccharide was extracted from* P. gingivalis* cultured according to the method described by Lee et al. [14].* E. coli* LPS was purchased from Sigma (B55:05, St. Louis, MO, USA).

### 2.4. Confocal Microscopy

hDPCs were seeded and cultivated in 2-chamber glass slides (Nunc, Roskilde, Denmark) for 12 h. After washing with PBS, cells were incubated in serum-free OPTI-MEM for an hour. Fluorescein isothiocyanate- (FITC-) labeled 1, 10, and 50 *μ*M of GV1001 peptides were added to cells and incubated for 2 h. The cells were fixed with 4% paraformaldehyde solution for 20 min at room temperature. Cells were stained with TO-PRO-3 Iodide 642/661 nm (Invitrogen, Grand Island, NY, USA) to visualize nuclei and were subjected to confocal microscopy. Colocalization of the peptides and nuclei was assessed using an FV1000 lager scanning confocal microscope (Olympus).

### 2.5. Western Blot Analysis

After addition of 0.75 *μ*M–10 *μ*M GV1001 to LPS-treated cells, the samples were prepared for electrophoresis and were separated using a 10% sodium dodecyl sulfate-polyacrylamide (SDS-PAGE) gel with a previously established buffer system [15]. After electrophoresis, the proteins were transferred onto a nitrocellulose membrane and blocked by Tris-buffered saline (TBST, 0.05% Tween-20) containing 5% nonfat dried milk. The membranes were then incubated with anti-p-ERK and anti-p-p38 (Cell Signaling Technology, Beverly, MA, USA) antibodies and subsequently washed with TBST. Antigen-antibody complexes were visualized using an enhanced chemiluminescent detection system (West-Zol, Intron, Seoul, Korea) by incubating membranes with goat anti-rabbit IgG or goat anti-mouse IgG antibody coupled to horseradish peroxidase (Santa Cruz Biotechnology, Inc., Santa Cruz, CA, USA), diluted at 1 : 2000. The blots were stripped and reprobed with an anti-*β*-actin polyclonal antibody to ensure that equal amounts of protein were used.

### 2.6. Real-Time RT-PCR

After treatment with 0.75–20 *μ*g mL^−1^ LPS for 2, 4, 6, and 8 h, total RNA was isolated from the cells using Trizol reagent (Life Technologies Inc., Gaithersburg, MD, USA) according to the manufacturer's suggested protocol and treated with DNase I (RNase-free, RQ1; Promega, Madison, WI, USA). One microgram of total RNA was used as a template to create first-strand cDNA with oligo-dT priming using an Omniscript RT Kit (Qiagen Inc., Valencia, CA, USA). The quantitative real-time PCR analyses were performed using an ABI Prism 7500 Real-Time PCR System (Applied Biosystems, Foster City, CA, USA) with a SYBR Premix Ex Taq II kit (Takara, Otsu, Japan) and 35 cycles of PCR. The denaturing, annealing, and extension conditions of each PCR cycle were 95°C for 10 s, 60°C for 15 s, and 72°C for 10 s, respectively. The relative amount or fold change of the target gene was normalized relative to the level of the control (untreated cells). The following primer sequences were used in the real-time PCR reactions: 5′-CGA AAG TCA ACT CCA TCT GCC-3′ and 5′-GGC AAC TGG CTG GAA GTC TCT-3′ for IL-6 gene; 5′-CCA GGA GAA AGT CAG CCT CCT-3′ and 5′-TCA TAC CAG GGC TTG AGC TCA-3′ for TNF-*α* gene; 5′-GTG GTG GAC CTG ACC TGC-3′ and 5′-TGA GCT TGA CAA AGT GGT CG-3′ for GAPDH gene.

### 2.7. Cytotoxicity Assay

To evaluate the cytotoxicity of GV1001 on hDPCs, cells (2 × 10^5^/well) were treated with several concentrations of GV1001 (0, 1, 5, 10, and 50 *μ*M/well) for 48 hours. The cells were incubated with 5.7 mol L^−1^ of MTT solution for 4 h in a tissue-culture incubator. A 200-lL quantity of dimethyl sulfoxide solution was then added to the cell-culture wells, and the plates were shaken for 10 min at room temperature to dissolve the precipitated formazan crystals. The solution was centrifuged for 10 min, and the optical density of the supernatant was measured at wavelength of 540 nm using an ELISA plate reader (PowerWave X 340; BioTek Instruments, VT). 0.9% NaCl solution was used as a negative control. The MTT assay was performed three times.

### 2.8. Statistical Analysis

The data are expressed as the mean ± standard deviation (SD) of at least 3 separate experiments. Comparisons between 2 groups were analyzed using Mann-Whitney *U* test. *P* values less than 0.05 were considered statistically significant.

## 3. Results

### 3.1. Internalization of GV1001 Peptide

**Figure 1:**
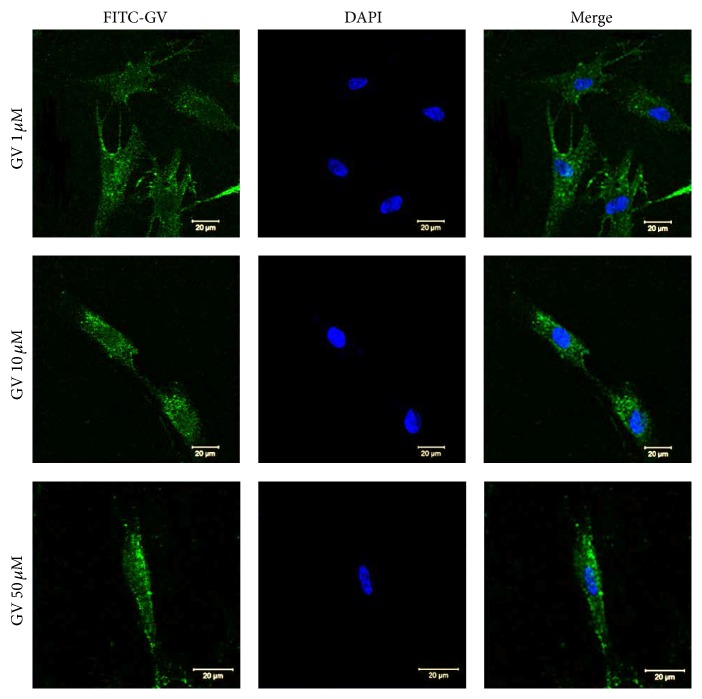
Internalization of various GV1001 concentration by hDPCs. 1, 10, and 50 *μ*M GV1001 peptides labeled with FITC at the C-terminus (GV1001-F) were used to treat hDPCs as described in Section 2. Internalization of the peptides was analyzed by confocal microscopy.

To confirm the cell-penetrating activity of GV1001, FITC was conjugated to the C-terminus of 1, 10, and 50 *μ*M GV1001 peptides (GV1001-F) and subjected to an internalization assay using confocal microscopy. As shown in Figure 1, GV1001-F showed mostly cytoplasmic distribution in hDPCs in various levels of peptide concentration.

### 3.2. LPS of* E. coli* and* P. gingivalis* Induced Expression of Inflammatory Cytokines in hDPCs

**Figure 2:**
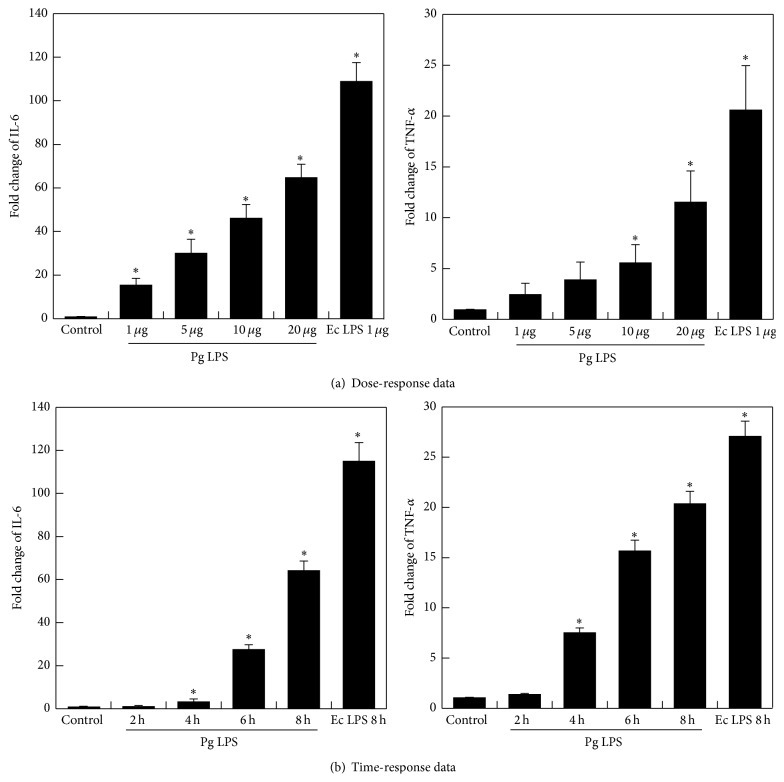
Effects of various doses of LPS and exposure times on the expression of TNF-*α* and IL-6 in hDPCs. hDPCs were serum-starved for 24 h and treated with indicated concentrations of* P. gingivalis* LPS (1, 5, 10, and 20 *μ*g mL^−1^) (a), for different times (2, 4, 6, and 8 hrs) (b). The levels of TNF-*α* and IL-6 mRNAs were determined by RT-PCR. Each value indicates the mean ± SEM of three independent experiments. *∗* indicates a significant difference (*P* < 0.05) relative to nontreated cells as control.* E. coli* LPS as positive control was treated 1 *μ*g mL^−1^.

To verify the effects of various doses of LPS on inflammatory cytokine expression in hDPCs, the levels of TNF-*α* and IL-6 expression were evaluated using real-time PCR after exposing hDPC cells to LPS of* P. gingivalis* or 1 *μ*g mL^−1^ of* E. coli* at concentrations of 1, 5, 10, or 20 *μ*g mL^−1^ for 10 hrs. The results showed that IL-6 transcript was profoundly induced in the presence of 1, 5, 10, or 20 *μ*g mL^−1^ of* P. gingivalis* LPS or 1 *μ*g mL^−1^ of* E. coli* LPS (*P* < 0.05), and TNF-*α* transcript was profoundly induced in the presence of 10 or 20 *μ*g mL^−1^ of* P. gingivalis* or 1 *μ*g mL^−1^ of* E. coli* LPS (*P* < 0.05) (Figure 2(a)).

To evaluate the effects of LPS on inflammatory cytokine expression in hDPCs over a period of time, the levels of TNF-*α* and IL-6 expression were evaluated using real-time PCR after exposing hDPCs to 20 *μ*g mL^−1^ *of P. gingivalis* LPS or 1 *μ*g mL^−1^ of* E. coli* LPS for 2, 4, 6, or 8 hrs. Upregulation was found in hDPCs after 4 to 8 hrs of exposure to 20 *μ*g mL^−1^ of* P. gingivalis* LPS or 1 *μ*g mL^−1^ of* E. coli* LPS (*P* < 0.05) (Figure 2(b)). The expression of TNF-*α* and IL-6 in hDPCs were upregulated by LPS in a time- and dose-dependent manner.

### 3.3. GV1001 Downregulated the Expression of TNF-*α* and IL-6 in LPS-Stimulated hDPCs

**Figure 3:**
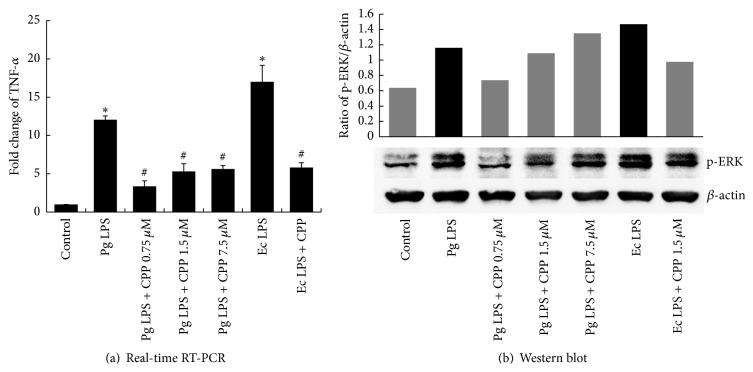
Effects of GV1001 on LPS-induced TNF-*α* and p-ERK production in hDPCs. 0.75, 1.5, and 7.5 *μ*M GV1001 were added to hDPSCs treated with 1 *μ*g mL^−1^ of* E. coli* LPS or 20 *μ*g mL^−1^ of* P. gingivalis* LPS for 10 hrs. RT-PCR and Western blot revealed downregulation of (a) TNF-*α* and (b) phosphorylated ERK, respectively, after exposure of cells incubated with GV1001 and 1 *μ*g mL^−1^ of* E. coli* LPS or 20 *μ*g mL^−1^ of* P. gingivalis* LPS. Each value indicates the mean ± SEM of three independent experiments. # indicates a significant difference (*P* < 0.05) relative to cells treated with LPS in the presence of GV1001.

**Figure 4:**
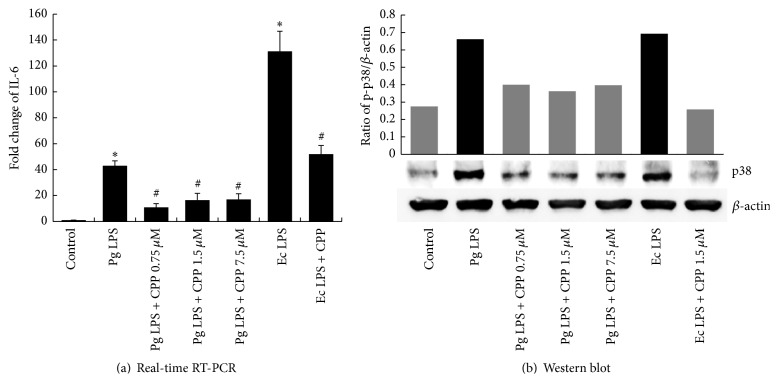
Effects of GV1001 on LPS-induced IL-6 and p-p38 expression in hDPCs. 0.75, 1.5, and 7.5 *μ*M GV1001 was added to hDPCs treated with 1 *μ*g mL^−1^ of* E. coli* LPS or 20 *μ*g mL^−1^ of* P. gingivalis* LPS for 10 hrs. Real-time RT-PCR and Western blot revealed downregulation of (a) IL-6 and (b) phosphorylated p38, respectively, after exposure to 1 *μ*g mL^−1^ of* E. coli* LPS or 20 *μ*g mL^−1^of* P. gingivalis* LPS incubated with GV1001 (0.75, 1.5 *μ*M, and 7.5 *μ*M). Each value indicates the mean ± SEM of three independent experiments. # indicates a significant difference (*P* < 0.05) relative to cells treated with LPS in the presence of GV1001.

We measured TNF-*α* and IL-6 expression after LPS stimulation to determine the anti-inflammatory activity of GV1001 peptide by real-time PCR. LPS-stimulated TNF-*α* and IL-6 production was significantly inhibited (*P* < 0.05) (Figures 3(a) and 4(a)).

### 3.4. GV1001 Effectively Suppressed the Activation of ERK and p38MAPK by LPS Stimulation

To investigate whether the anti-inflammatory effect of GV1001 is mediated by the suppression of ERK and p38MAPK activation, regulation of phosphorylated ERK and phosphorylated p38MAPK was evaluated by Western blot analysis. As shown in Figures 3(b) and 4(b), GV1001 decreased LPS-induced phosphorylation of ERK and p38MAPK.

### 3.5. GV1001 Has No Cytotoxic Effect on hDPCs

**Figure 5:**
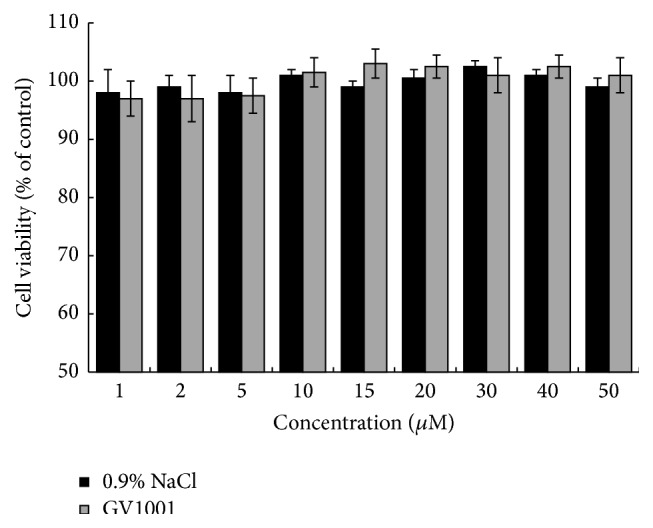
Effects of GV1001 on viability of hDPSCs. The cells (2 × 10^5^ cells/well) were treated with the indicated concentrations of GV1001 for 48 h. The cell viability was assessed by an MTT assay and the surviving cell values are shown as a percent of the control-treated cells (no addition of GV1001); 0.9% NaCl solution was used as a negative control. Each value indicates the mean ± STDEV of three independent experiments.

hDPCs were exposed to GV1001, and cytotoxicity was tested by MTT assay. Incubating GV1001 peptide with hDPCs for 48 h did not affect cell viability in all concentrations tested (0–50 *μ*M), which showed a pattern similar to that seen with 0.9% NaCl (*P* < 0.05) (Figure 5).

## 4. Discussion

Although endodontically treated teeth can maintain their function for a prolonged period of time, there are many advantages to maintaining pulp vitality. In immature permanent teeth with incomplete apical and dentinal wall development, reparative dentin formation is critical for further development of the teeth. Maintaining the vital pulp also has the benefit of reducing the occurrence of apical periodontitis by blocking bacterial infections [16]. Based on these advantages, it is preferable to maintain or renew pulp vitality rather than use current endodontic therapies. Recently, successful pulp regeneration and revascularization techniques have been developed and are gaining popularity.

*P. gingivalis* is an obligately anaerobic, Gram-negative bacterium that has been positively associated with destructive periodontal disease [17, 18]. It is one of the most pathogenic species among black-pigmented Gram-negative anaerobes [19]; it is frequently present in root canal infections and other odontogenic abscesses of patients not suffering from periodontal disease, which confirms its pathogenic role [20].* P. gingivalis* may also play a role in symptomatic infections, such as acute apical abscess [21, 22]. Furthermore, there is a positive association between* Porphyromonas* spp. and pain, mechanical allodynia, swelling, and purulent exudates in root canals [22, 23].

Pathogens are generally detected by specific receptors, one of which is the toll-like receptor (TLR) family. TLR stimulation initiates host defense through the activation of several intracellular signaling pathways, including activation of the mitogen-activated protein (MAP) kinases [24]. Exposure of cells to inflammatory stimuli, including LPS and proinflammatory cytokines, results in phosphorylation of p38MAPK [8].

Once the TLR is stimulated by a pathogen, proinflammatory cytokines and chemokines are produced by the odontoblast, resulting in recruitment and stimulation of immune effector cells and also direct bacterial killing [25]. Although these biological responses protect the host against invading pathogens, the inflammatory response also leads to host tissue damage. Hence, regulating the production of these cytokines is pivotal to protecting host tissue.

The extra-telomeric functions of hTERT are suggested regarding cellular proliferation, stem cell mobilization, and antiapoptotic, antiaging, and antioxidant effects through mitochondrial stabilization, transcriptional regulation [26, 27]. If hTERT peptide treatment that mimics the extra-telomeric functions of hTERT could be developed, it could be used in the treatment of pulpal diseases. In this study, the potential use of a small peptide of human origin to control pulpal inflammation as a therapeutic agent was investigated. The anti-inflammatory effect of GV1001 is achieved by modulating the suppression of the activation of ERK and p38 MAPK and the subsequent cytokine production induced by* P. gingivalis* LPS stimulation.* P. gingivalis* and* E. coli* LPSs induced TNF-*α* and IL-6 expression in hDPC in a time- and dose-dependent manner, while GV1001 peptide downregulated inflammatory cytokines, IL-6 and TNF-*α*. hDPCs expressed TLR2 (data are not shown) and upregulated phosphorylated ERK and p38 MAPK in response to stimulation by LPS. From the similar suppression patterns of the two, it could be assumed that the downregulation of inflammatory IL-6 and TNF-*α* by GV1001 was dependent on p38 MAPK and ERK-signaling, respectively, thus indicating that MAPK signaling was required in downregulation of inflammatory cytokines in dental pulp stem cells by GV1001 peptide. As shown in Figure 3, higher dose of GV1001 exhibited a lower effect on TNF*α* induced by* P. gingivalis* LPS. One of the possible reasons is that GV1001 can partially aggregate in the high concentration in cell culture medium because cell culture medium does not include carrier protein. Thus, the lower concentration of GV1001 except aggregating GV1001 penetrates into the cells and reacts with signaling molecules related with inflammation.

In another previous study, GV1001 peptide was reported to have the ability to penetrate into various cells, including cancer cell lines and primary blood cells, without affecting cell viability [13]. GV1001 was predominantly located in the cytoplasm and was used to successfully deliver macromolecules such as proteins, DNA, and siRNA into cells [13]. These cell-penetrating peptides (CPPs) have become one of the most popular and efficient tools for delivering various molecules into cells owing to the fact that they have the ability to enter cells independently of a membrane receptor, and they show no cell-type specificity [28]. This study is significant in that it is the first to demonstrate GV1001 peptide as a CPP of human origin with a self-anti-inflammatory effect and without affecting cell viability.

The use of GV1001 peptide can be a potential therapeutic approach for treating pulpal inflammation and the peptide can also be used as an intracellular delivery tool for bioactive molecules. Using GV1001 peptide as a pulp-capping agent on reversibly inflamed pulp or an alternative to antibiotics in regeneration therapy may be effective. Conjugating growth factors such as TGF-*β*s and BMPs with GV1001 can facilitate induction of hDPCs to differentiate effectively into odontoblast-like cells. Moreover, gene therapy can be implemented using GV1001 to fuse a growth/differentiation factor for application in vital pulp therapy, regenerative endodontic fields and tissue engineering. The results from this study may support further research on the GV1001 peptide and its various clinical applications.

## 5. Conclusion

GV1001 has the ability to penetrate into the cell. The downregulation of LPS-induced TNF-*α* and IL-6 expression was mediated through ERK and p38 MAP kinase pathways. These findings provide mechanistic insight into the ability of GV1001 peptide to cause anti-inflammatory actions in LPS-stimulated pulpal inflammation without significantly affecting cell viability.
